# KIT mutations and expression: current knowledge and new insights for overcoming IM resistance in GIST

**DOI:** 10.1186/s12964-023-01411-x

**Published:** 2024-02-27

**Authors:** Shishan Zhou, Omar Abdihamid, Fengbo Tan, Haiyan Zhou, Heli Liu, Zhi Li, Sheng Xiao, Bin Li

**Affiliations:** 1 Division of Oncology, Xiangya Hospital, Central South University, Changsha, Hunan China Xiangya road 87#,; 2 Garissa Cancer Center, Garissa County Referral Hospital, Kismayu road, Garissa town, P.O BOX, 29-70100 Kenya; 3 Division of Surgery, Xiangya Hospital, Central South University, China Hunan Changsha,; 4 Division of Pathology, Xiangya Hospital, Central South University, Changsha, Hunan China; 5 Center for Molecular Medicine of Xiangya Hospital, Collaborative Innovation Center for Cancer Medicine, Central South University, Changsha, Hunan China 410008; 6 Department of Pathology, Brigham and Women’s Hospital, Harvard Medical School, Boston, 410008 MA USA

**Keywords:** KIT mutations and expression, Gastrointestinal stromal tumor, Therapeutic targets, Imatinib resistance

## Abstract

Gastrointestinal stromal tumor (GIST) is the most common sarcoma located in gastrointestinal tract and derived from the interstitial cell of Cajal (ICC) lineage. Both ICC and GIST cells highly rely on KIT signal pathway. Clinically, about 80-90% of treatment-naive GIST patients harbor primary KIT mutations, and special KIT-targeted TKI, imatinib (IM) showing dramatic efficacy but resistance invariably occur, 90% of them was due to the second resistance mutations emerging within the KIT gene. Although there are multiple variants of KIT mutant which did not show complete uniform biologic characteristics, most of them have high KIT expression level. Notably, the high expression level of KIT gene is not correlated to its gene amplification. Recently, accumulating evidences strongly indicated that the gene coding, epigenetic regulation, and pre- or post- protein translation of KIT mutants in GIST were quite different from that of wild type (WT) KIT. In this review, we elucidate the biologic mechanism of KIT variants and update the underlying mechanism of the expression of KIT gene, which are exclusively regulated in GIST, providing a promising yet evidence-based therapeutic landscape and possible target for the conquer of IM resistance.

Video Abstract

**Supplementary Information:**

The online version contains supplementary material available at 10.1186/s12964-023-01411-x.

## Summary

Imatinib resistance is the major obstacle for the cure of GIST, mostly due to second mutations within KIT gene for its reactivation. Hence, drug development during the past decades was focused on kinase inhibitors, aiming to broader the spectrum of KIT kinase mutations effectively inhibited, including sunitinib, regorafenib and ripertinib, this benefit, however, is modest compared with imatinib. The reason is the emergence of secondary resistant mutations showing highly heterogeneous, and these TKIs are active against only a subset of the KIT secondary mutational spectrum, which constitutes the main determinant for treatment failure in imatinib-resistant GIST. However, one of the most important characteristics of GIST cells in both treatment-naive or TKIs-resistant cells is that its high dependency on KIT signal that necessitate the recapitulation of the underlying biology or mechanism of KIT mutations and expression, with the purpose of developing novel drugs, alone or combined with current KIT-TKIs, to prevent or reverse resistance via the complete elimination of KIT oncogenesis.

## Introduction

The tyrosine kinase(TK) KIT was described for the first time in 1987 as the human cellular homologue of the feline sarcoma viral oncogene v-kit [1]. It located on chromosome 4q12 and contains 976 amino acids, which encodes a transmembrane protein belonging to the type III family of RTK (receptor tyrosine kinases) [2, 3]. The extracellular segment contains five immunoglobulin-like structural domains (D1-D5), of them D1-D2-D3 are stem cell factor (SCF) binding regions, and D4-D5 are important units for KIT dimer formation [4]. KIT is a transmembrane glycoprotein with ligand-induced TK activity [5]. Its ligand SCF was identified in 1990 and it exists both as a membrane-bound and soluble form [6]. Upon binding of SCF, dimerization of neighboring KIT receptors is mediated by homotypic interactions at the D4-D5 interface. This is followed by asymmetric arrangements of the cytoplasmic region of the KIT dimers associated to trans-autophosphorylation and final kinase activation [7]. Physiologically, in addition to interstitial cells of Cajal (ICC), KIT receptor is expressed in germ cells, bone marrow stem cells, melanocytes, and mast cells. In response to SCF stimulation, KIT propagates the signal of survival and proliferation to ICC, maintaining the function of gut motility [8–10].

Gastrointestinal stromal tumor (GIST) is the most common mesenchymal neoplasm located in the gastrointestinal tracts. The pathogenesis of GIST involve gain-of-function mutation in KIT (accounts for 75–80%), platelet-derived growth factor receptorα(PGFRA) (< 10%), or gene abnormalities including succinate dehydrogenase (SDH)-deficiency, the mutations of neurofibromin 1 (NF1), Kirsten rat sarcoma viral oncogene homolog (KRAS) and Harvey rat sarcoma viral oncogene homolog (HRAS) pro-oncogenes [11–13]. GIST cells arise from the musculature of ICC or their precursor cells and highly rely on the KIT expression for survival [14, 15]. KIT mutations is an early step for the development of GIST from ICC, meaning KIT necessitates the survival of GIST cells [16]. KIT mutations, usually occur at the intracellular juxtamembrane (JM) WW domain (encoded by exon 11), or in the membrane-proximal extracellular domain (encoded by exons 8 or 9), accounting for approximately 85–90% of KIT-mutant GIST, they endows KIT gene an oncogenic capacity to proliferate and form tumors via the intermediates of PI3K-AKT, JAK-STAT and RAS-RAF-MEK-ERK (MAPK) cascades [9, 12, 17–19].

With the discovery of this druggable KIT mutations, KIT-targeted inhibition with first line Imatinib (IM) become standard of care and offers meaningful clinical benefit in metastatic GIST patients [14, 20]. However, the primary KIT variants showing different sensitivity to IM, treatment resistance is common and occurs between 18–24 months of imatinib treatment due to resistant clones, warranting a switch to second and beyond-second lines of tyrosine kinase inhibitors (TKIs) including sunitinib, regorafenib and repritinib as per the guideline, but only with modest efficacy [21–23]. Furthermore, almost all of the patients will progress presented with the metastasis to multiple organs and each of these metastases, or even in the same primary lesion can have different genomic mutations with KIT [24, 25]. These range from point mutations or insertions to large indel variants, which exhibit variable biological traits, including intracellular mis-location, and downstream target molecules, which possibly as the determinants to the response to IM [26, 27]. Moreover, the sole transcription factors were identified in the regulation of these KIT mutants, showing much more different from that of wild type (WT) KIT [28, 29]. These distinct regulation of KIT in GIST could also be causative for its blocked degradation. In fact, KIT expression was observed in 95% of GIST, as a diagnostic algorithm using upfront immunohistochemistry (IHC) markers of KIT (CD117) positivity in GIST [30], and with IM treatment, KIT transcript or/ and protein derived from multiple heterogeneous KIT mutations in IM-resistant cells is higher than that in pre-IM counterparts [31–33], implying the compensatory up-regulation of KIT possibly contributing to secondary resistance to IM. Here, we summarize various of KIT variants and its core regulated network, focus on the process of gene regulation, transcription and protein translation, with emphasis on the therapeutical vulnerability and clinical strategy for targeting oncogenic KIT kinase dependency in GIST.

## KIT mutations and expression in GIST

### KIT mutations

Within the larger group of KIT-mutated GIST, different mutations’ hotspots have been reported, of which the vast majority of KIT mutations are found in exon 11 coding for JM (66–71%), exon 9 coding for extracellular domain (13%), exon 13 coding TK domain I (ATP binding pocket) (1–3%), and exon 17 coding for TK domain II (activation loop) (1–3%). The ATP-binding pocket, encoded by exon 13 and 14, whose mutations directly interfere with IM binding, or the activation ring, where mutations can stabilize the KIT's active conformation [12, 34]. The gain-of-function of KIT mutations resulting in the constitutive activation of the protein, genomic context and alternative signaling pathways, also largely affect the efficacy of IM, as well as sunitinib, and regrifinib and ripritinib, which mostly due to the different transcriptional programs observed in GIST with various genotypes that influenced the protein active structures, dimerization affinity, and cellular localization [35]. Indeed, clinically, most GIST show strong, diffuse cytoplasmic staining, whereas nearly half show concurrent dot-like (bGolgiQ pattern) staining and occasionally, only dot-like or even membranous staining is seen [36, 37]. In vitro GIST cell lines, different KIT mutations were detected, such as classical GIST-T1 (primary mutation in KIT exon 11—Δ560–578) and GIST-882 (primary mutation in KIT exon 13—K642E) cell models, the biological effects upon imatinib treatment can be significantly different [38–40]. Notably, as shown in Table 1, the involved down-streams varied across different KIT genotypes, and which also be compelling factors impact tumor behavior and IM sensitivity.

**Table 1:** KIT mutation and its activated downstream in GIST cell lines

Cell lines	KIT mutations	Mutation downstream	TKI resistance and IC50 (nM) IM SU RE	Ref
GIST-T1^+^	Exon 11: V570-Y578 (Hom)	MAPK pathway PI3K pathway Wnt pathway Notch pathway STAT3	S S S 16.54 15.00 110.00	[41–46]
GIST-882^+^	Exon 13: K642E (Hom)	MAPK pathway PI3K pathway STAT3	R S R 173.00 54.00 503.00	[19, 40, 44, 47, 48]
GIST-48^+^	Exon 11: V560D (Hom) Exon 17: D820A (Het)	MAPK pathway PI3K pathway JAK/STAT pathway	R R R 413.00 587.00 164.00	[44, 47, 49, 50]
GIST-430^+^	Exon 11: (V560-L576) (Het) Exon 13: V654A (Het)	MAPK pathway PI3K pathway	R R R 61.00 68.00 191.00	[44, 49–51]
GIST-BOE^+^	Exon 9: A502_Y503dup	MAPK pathway	R NR NR NR	[52, 53]
GIST-PSW^+^	Exon 11: K558_G565delinsR	MAPK pathway	S NR NR NR	[52, 54]
HG129^+^	Exon 11: 45 bp insertion between F591- 592G	MAPK pathway	S NR NR 42.00 NR NR	[55]
GIST-522^−^	Exon 11: delEVQWK554-558 (het)	NR	R NR NR NR	[49]
GIST-62^−^	Exon 11: MYEVQWK552-558T (het)	NR	R NR NR NR	[49]
GIST-226^−^	Exon 11: P551-W557 (Hom) Exon 17: Y823D (Hom)	MAPK pathway	R R R > 5000.00 3856.00 > 5000.00	[44, 56]
GIST-48B^−^	Exon 11: V560D (Hom) Exon 17: D820A (Het)	MAPK pathway	R R R > 5000.00 > 5000.00 > 5000.00	[44, 57]
GIST-5^−^	Exon 11	NR	NR NR	[58]
GIST-474^−^	Exon 11	NR	NR NR	[58]
HG209^−^	Exon 11: delYIDPTQL 570–576 Exon 17: D816H	MAPK pathway	R R NR > 1000.00 NR NR	[59]
GIST-544^−^	Exon9: AY503-504ins (Het)	MAPK pathway STAT1 and STAT3	NR NR	[60]

*IM* Imatinib, *SU* Sunitinib, *RE* Regorafenib, *Hom* Homozygous, *Het* Heterozygous, + KIT positive,—KIT negative, *S* Sensitive, *R* Resistance, *NR* Not reported

### KIT expression

**Fig. 1:**
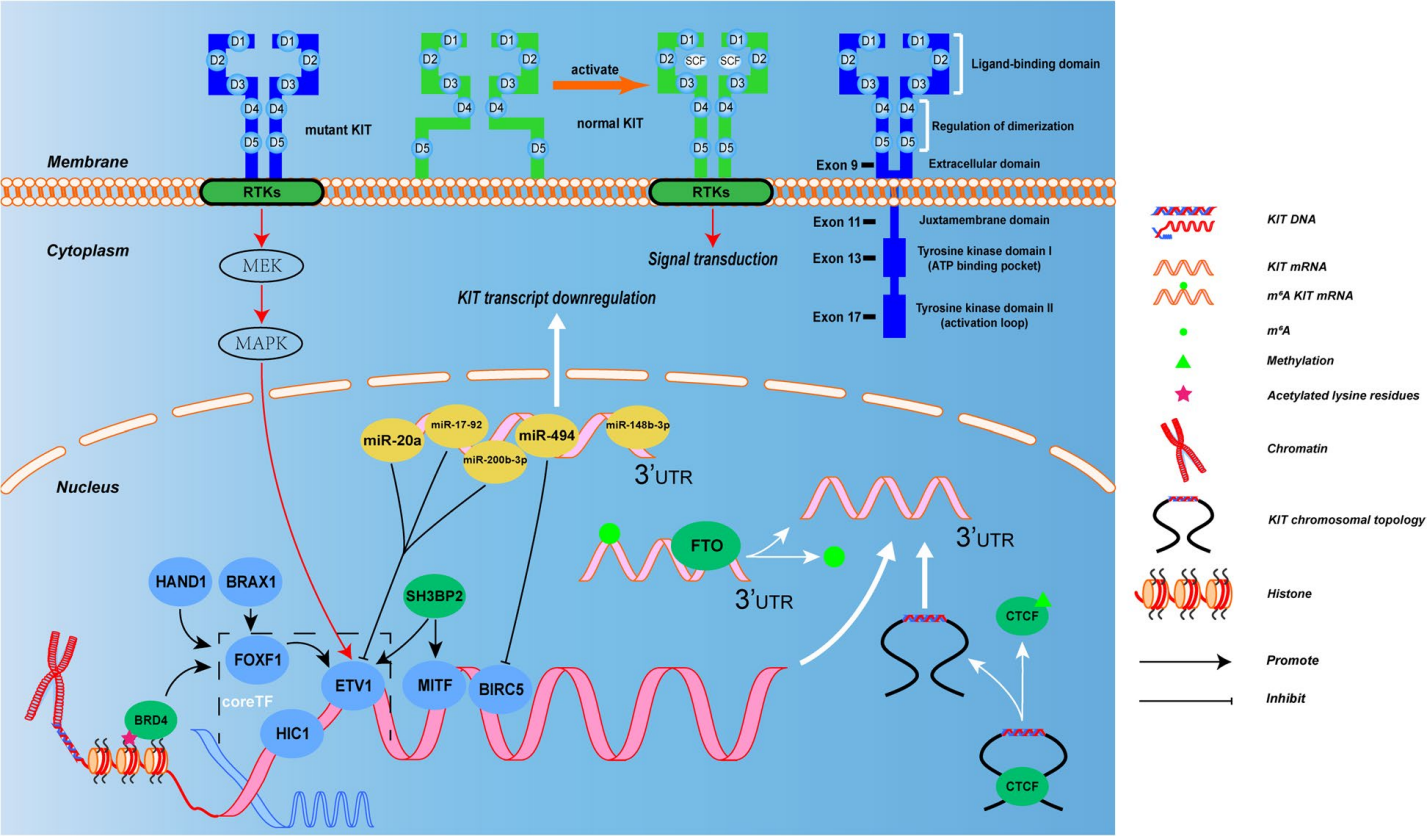
Gene and epigenetic regulations of KIT expression in GIST. KIT extracellular segment contains five immunoglobulin-like structural domains (D1-D5), D1-D2-D3 are stem cell factor (SCF) binding regions, and D4-D5 are important units for KIT dimer formation. In the absence of ligand stimulation, two adjacent KIT protein D4 structures remain independent due to charge repulsion. KIT mutations usually arise in the extracellular domains (exon 9), the juxtamembrane domain (exon 11) and in the kinase I and II domains (exons 13 and 17). The SCF binding to KIT and then changes the conformation of KIT and promote its dimerization, thereby activating tyrosine kinase activity in the intracellular segment by recruiting and phosphorylating substrates, thus forming signal transduction. But once KIT mutated, it disrupts its self-inhibition mechanism, resulting in continuous activation of signaling pathways such as Ras-Erk within the cell. FOXF1, ETV1 and HIC1 together form the core TF network in GIST binding enhancer and/or promoter and then promoting KIT expression. BRD4 (function as “readers”), HAND1 and BRAX1 positive regulate the core TF network. Transcription factor MITF and BIRC5 promote KIT transcription. MiRNA-20a, miRNA-17–92, miRNA200b-3p, miRNA-494, miRNA-148-3p are tumor suppressors downregulate KIT transcription and the majority of them directly bind to the 3’-UTR domain of relevant mRNA; the first three mentioned miRNAs can inhibit ETV1 mRNA levels, miRNA-494 can inhibit BIRC5 expression suggesting a negative feedback mechanism. SH3BP2 promotes the expression of mutant KIT by up-regulation of the expression of MITF and ETV1. FTO, functions as a “eraser”, promoting m6A demethylation increases the expression of KIT. Normal KIT chromosomal has CTCF insulator creating a topological boundary. Once CTCF insulator displaced by DNA methylation, allowing the super-enhancer to contact and induce KIT

Generally, DNA expression is regulated by the cis-regulatory elements (CREs), which consists of enhancers and super-enhancers transcription factors which comprise of transcription factors and the recruited co-activators and RNA Polymerase II (RNA Pol II). Over-expression of KIT in GIST results from the dysregulation of a large enhancer domain in the DNA strand, but rarely related to its DNA amplification [61]. CHIP-seq of GIST tumor samples and cell lines identified the enhancer domain to be driving KIT gene expression, which is unique and essential for KIT gene expression and cell viability in GIST. Accordingly, exclusive transcription factors model the expression of GIST-type mutant KIT gene which facilitates tumorgenesis and progression [62]. Moreover, GIST cells are highly dependent on KIT expression, which was due to epigenetic regulation rather than amplification in KIT oncogene [61]. However, as more epigenetic mechanisms elucidated in mesenchymal tumors, reversible epigenetic changes can be identified and appears to be an important novel approach to understand the formation, prognosis and therapeutic strategies of various types of mesenchymal tumors [63–65]. Figure 1 shows KIT mutations and expression in GIST and its role in epigenetics and multifaceted biological modifications.

### Regulation of KIT coding gene

The regulations of gene are mainly connected with transcription factor (TF), which directly interpret the genome, and no exception in the change of KIT gene [35, 66, 67]. It seems that TFs forming the regulatory network exclusively act on the enhancers of KIT gene in GIST, showing its rudiment [62, 68–70]. In addition, the study of how TF linked to essential chromatin regulators will also provide important insights into the gene regulation and epigenetic mechanisms of GIST [71].

Forkhead box F1 (FOXF1) is a member of the forkhead box (FOX) family, which contains a highly conserved DNA binding region (DBD) [72]. As one of the key TFs, FOXF1 is required for the lineage differentiation of ICCs, as well as the growth of GIST cells. FOXF1 functions as a pioneer factor that modulates the chromatin accessibility for ETS translocation variant 1 (ETV1), after which they accumulate in the enhancer domain of the mutant KIT gene, promoting KIT expression [73, 74]. ETV1, an ETS family transcription factor, is located on chromosome 7p21 [75]. It is involved in the tumorigenesis of multiple cancer types, including prostate cancer and melanoma, where it regulates distinct transcriptional programs [76]. ETV1 is also a master regulator of the ICC lineage, and it is essential for the development of the subtypes of ICCs which are sensitive to oncogenic KIT-mediated transformation. Moreover, ETV1 directly binds to the enhancer and promoter regions of KIT gene and thus forming a positive feedback regulation that advances their expression [70]. Notably, the protein of ETV1 is highly unstable, and active mitogen-activated protein kinase (MAPK) can increase its stability. KIT mutants activate the downstream, MAPK, leading to the stabilization of the ETV1 protein and oncogenic ETS transcript program. Co-activators cyclic AMP-responsive element-binding protein (CREB)-binding protein (CBP) and p300 interact with an oncoprotein ETS translocation variant 1(ETV1), which directly acetylates ETV1, thus enhancing its stability, DNA-binding capacity, and transcriptional activity in vitro [77].

This mechanism of ETV1 in GIST differs from that in the other ETS-dependent tumors, such as genomic translocation or amplification in prostate cancer [78]. FOXF1 loss results in decreased ETV1 protein, and global loss of ETV1 chromatin binding. Therefore, FOXF1 inhibition causes the reduction of KIT approximately by 2-to-eightfold than ETV1 inhibition does, while ETV1 knockdown showed no impact on FOXF1 expression. These data indicate that the regulation of FOXF1 in GIST may be pre-determined in ICCs precursors [73].

Heart and Neural Crest Derivatives Expressed 1 (HAND1) is a key TF involved in the placentation and morphogenesis of the heart [79], and its deficiency during embryogenesis can cause congenital cardiac defects or adult heart failure [80]. Similarly, HAND1 is involved in the transcriptional amplification of the KIT oncogene via its influence on the expression or protein interactions of the core TF network of KIT [35], including ETV1, HIC1 (hypermethylated in cancer 1), FOXF1 and other GIST-correlated protein, and G protein-coupled receptor 20(GPR20) [81]. Physiologically, Homeobox protein BarH-like 1 (BARX1) controls the development of gastric smooth muscle and spleen, as well as intestinal rotation [35]. Similar to HAND1, BARX1 is a TF localized in the nucleus, which is closely correlated with indolent and micro-GIST, albeit the underlying mechanism remaining unclear [82].

The c-Abl Src homology 3 domain-binding protein-2 (SH3BP2) has 561 amino acids, containing an SH3-binding proline-rich region, an N-terminal pleckstrin homology (PH) domain, and a C-terminal SH2 domain [83, 84]. It serves as a cytoplasmic adaptor protein, which is generally expressed in GIST cells. The epigenetic silencing of SH3BP2 leads to decreased oncogenic KIT and PDGFRA expression at both mRNA and protein levels [84]. SH3BP2 promotes the expression of mutant KIT via up-regulation of the expression of microphthalmia-associated transcription factors (MITF) and ETV1. Conversely, KIT can expedite the expression of SH3BP2 and MITF, demonstrating a positive feedback loop that exists in these molecules [85]. Silencing of SH3BP2 induces miRNAs (miR-1246 and miR-5100) expression which targets MITF and ETV1, thus decreasing KIT expression [86]. A recent study found that reconstitution/recombination of MITF restored KIT expression levels in SH3BP2-silenced cells and restored cell viability in mesenchymal tumors, while also reducing MITF and ETV1 expressions [87]. Meanwhile, SH3BP2 silence can also attenuate PI3K activation induced by KIT kinase [84]. In GIST, TFs such as HAND and the core TF network constitutes a large positive feedback system for KIT mutant gene expression, and this positive feedback promotes the development of mesenchymal tumors. The common regulatory mechanism of these transcription factors in the regulation of KIT-encoding genes is unclear warranted to be further investigated.

### Epigenetic modification

Epigenetic modifications are closely associated with genome-wide transcriptional regulation in cancer, and it has also been extensively studied in GIST pathogenesis, including progression and drug resistance. It refers to a stable heritable change in biological phenotype or gene expression without a change in nucleotide sequence [88, 89]. The key molecular mechanisms of epigenetic modification of DNA and chromatin can be divided into four main categories: DNA and RNA methylation [90, 91]; non-coding RNA; covalent post-translational histone modifications and non-covalent mechanisms [92]. All epigenetic changes and regulation are mediated through epigenetic enzymes (EEs), the functions of which can be divided into three categories: writers (responsible for modifications); erasers (remove modifications); and readers (identify and direct these modifications to the correct location) [93]. In KIT-mutated GIST, chromosomes are frequently lost at 1q, 13q, 14q, 22q, etc., involving Protein phosphatase 1A (PPM1A), kinesin family member 1B (KIF1B) and neurofibromin 2 (NF2) gene, but this loss does not occur in WT GIST, which show the alternative epigenetic alterations [28]. Therefore, in this section we explore the role of several epigenetic modifications in relation to KIT and the possible implications for future development.

### DNA and RNA methylation

DNA methylation is generally the 5-methylcytosine that occurs on the CpG islands [94] and is often described as a "silent" epigenetic marker [91] that plays important role in the maintenance of imprinting, genomic stability, development, and gene regulation in cancer [63, 95, 96]. Endoglin (ENG, CD105), a transmembrane glycoprotein expressed on activated vascular endothelium and other cells, is a co-protein of the transforming growth factor-h (TGF-h) receptor system. High expression of the ENG gene has been demonstrated in human as well as in mouse model of GIST[96, 97]. And the elevated ENG expression in KIT oncogenic mutants appears to be indirectly mediated by DNA hypomethylation, but the mechanism of this DNA methylation regulation is not fully understood [98]. The DNA-binding insulator protein CCCTC-binding factor (CTCF) and cohesion define the boundaries of chromosomal domains, also called topologically associated domains (TADs) [99–101]. It is reported that the super-enhancer was shared by Anoctamin 1(ANO1), which encodes the GIST clinical biomarker also known as DOG-1 (‘Discovered on GIST-1’) and FGF3/4, which reside in a ~ 250 kb TAD flanked by boundaries that contain CTCF binding sites, and the adjacent TAD on the 11q side contains a large cluster of super enhancer (SE), was recently observed in SDH-defcient GIST. In SDH-defcient GIST, DNA CpG methylation replaced CTCF binding in the FGF and KIT locus leading to abnormal contact between the starter switch and oncogenes [102]. So, it is postulated that in GIST, continuation of IM-based therapy for IM-resistant GIST might facilitate disease progression by promoting the malignant behavior of tumors in an FGF2-dependent manner.

Epi-transcriptional modifications are emerging as promising therapeutic targets in cancers. The most important classification of RNA methylation is the modification of RNA [103], including N6-methyladenosine (m6A) and 5-Methylcytosine, etc. [104]. As the most important classification of RNA methylation, the mechanism of M^6^A modification has been described in the development and treatment of different tumors, such as liver cancer, breast cancer and non-small cell lung cancer [105–107]. N^6^-methyladenosine of messenger RNA (mRNA), mainly occurring around the coding region or the stop codon of the 3´UTR, is the most abundant internal modification in mRNA. The levels of methyltransferase-like 3 (METTL3) is elevated and confer to Imatinib resistance in GIST patients, because METTL3 mediate the m^6^A modification on the 5’UTR of the multidrug transporter MRP1 mRNA, which facilitating MRP1 mRNA translation [108]. Besides, METTL3-mediated N6-methyladenosine (m^6^A) modifications facilitate miR-25-3p maturation which progression of GIST [109]. The fat mass- and obesity-associated (FTO) gene, termed as obesity-related gene, is located on chromosome 16q12.2 and is repurposed as a mRNA N^6^-methyladenosine (m^6^A) demethylase. A series of small-molecule compounds targeting FTO is developing therapeutic option in acute myeloid leukemia (AML) [110].

### Histone modification

Epigenetic modifications of histones, including methylation, phosphorylation, ubiquitination and acetylation, are the key processes for genes expression in GIST oncogenesis. Lysine (K)-specific demethylase 4D (KDM4D), as a histone demethylase, is overexpressed in GIST and promotes the progression of GIST via HIF1β/VEGFA signaling [111]. Bromodomain-containing protein 4 (BRD4) is a member of bromodomain and extra-terminal domain-containing (BET) family that include the proteins of BRD2, BRD3, BRD4, and BRDT. These proteins function as “readers” that tether acetylated lysine residues to both histone and non-histone proteins such as TF, and control genes expression. Accumulating evidences showed that BRD4 can regulate the nuclear factor kappa-light-chain-enhancer of activated B cells (NFκB) signaling, as a determinant in the progression of various cancers [112]. In GIST, BRD4 binds the acetylated lysine in histones or TF in enhancer or recruited pTEFb to initiate NFκB-dependent acetylated histones, promoting the transcription of c-KIT and C–C motif chemokine ligand 2 (CCL2), which is a chemokine recruiting macrophages to tumor functions as an immunosuppressor [113].

Monocytic zinc finger (MOZ) is a histone acetyltransferase that mediate the activation of histone acetylation in a complex with other molecules [114]. Using Genome-scale CRISP screening, the chromatin modifying enzymes, KAT6A/MOZ and KMT2A/MLL1 was found to be co-dependent and co-localized with GIST-associated genes and regulate oncogenic transcription and cell cycle progression by regulating transcription factor gene expression programs [115, 116]. KMT2A/MLL1 combined with Menin-MLL complex, is responsible for H3K4 methylation and transcription activation. Small molecules against MOZ or Menin remarkably reduced GIST tumor growth with synergistic toxicity in vivo and in vitro with the combination of KIT inhibitors [117].

### Non-coding RNAs on KIT gene expression

Non-coding RNAs (ncRNAs) are functional RNAs that is not be translated to proteins but as messager RNAs(mRNAs) functioning as regulators [118]. Micro-RNAs (miRNAs), a subtype of ncRNAs, are small endogenous RNAs of 19 ~ 22 nucleotides (nt) that regulate post-transcriptional silencing of target genes, usually in the 3′UTR [119]. Long non-coding RNAs (lncRNAs) are another important ncRNAs with more than 200nt transcripts. LncRNAs can fine-tune the expression of neighbor genes in a distinct context-dependent way via multifaceted mechanisms [120]. The largest study on miRNAs on primary tumors and metastases highlighted perpetuation of miRNAs features in metastatic lesions and that the primary origin appears to be the main determinant of the metastases miRNA profile [121].

### miRNAs

miR-218 which is a tumor suppressor directly bind to the 3’-UTR domain of relevant mRNA in lung cancer [122]. Similarly, in KIT-mutant GIST cells, miR-218 can exert its silencing effect on KIT expression by the direct bond to the 3′UTR of the KIT mRNA [123]. The Imatinib-resistant cell line, GIST430, can be re-sensitized to imatinib when the cells are transfected with miR-218, which possibly targets the PI3K/AKT pathway, KIT, or/and STAT3 molecules [124, 125]. MiR-148b-3p was largely described in the constraints of neoplastic transformation [126]. By binding to the nucleotides 1378–1393 and 1639–1656 of the 3’-UTR of KIT mRNA, miR-148b-3p can directly down-regulate the level of KIT transcript. However, a negative feedback loop causing KIT overexpression with an unknown mechanism offset the inhibitory role of miR-148b-3p. Therefore, miR-148b-3p alone shows no impact on the GIST cells, although it can synergize with IM to suppress the invasion and proliferation of GIST cells[48]. The correlation between downregulation of miRNA-221/222 and KIT expression was reported in several studies, indicating a tumor suppressor-miR in GIST [127, 128]. In vitro, the over-expression of miRNA-221/222 causes cell cycle arrest, apoptosis induction, and the inhibition of cell proliferation via decreasing KIT expression in erythroleukemic cells and erythropoiesis [129]. It binds to 3’UTR of KIT mRNA, where the rs17084733 variant interrupts the binding site of miR-221/222 in GIST cells [47, 130]. In addition, the expression of other miRNAs, including miR-142-5p [131], miR-9, miR-370, miR-494, and miR-501 [132], was negatively associated with the expression of the KIT gene in GIST cells*.* MiR-494 overexpression caused KIT downregulation and the reduction of its downstream p-AKT and p-STAT3 proteins via decreasing the expression of survivin (BIRC5), an important TF for KIT, which phenotype the KIT inhibition in GIST 882 [124, 133]. Additionally, miR-17–92, miR-200b-3p and miR20a cluster strongly downregulate the mRNA levels of the *ETV1*, which is the key TF in the regulation of the *KIT* gene [134, 135].

### LncRNAs

The lncRNA coiled-coil domain-containing 26 (CCDC26) was identified as a retinoic acid-dependent modulator and plays an important role in the pathogenesis of glioblastoma [136]. CCDC26 can inhibit cell proliferation via directly decreasing the KIT expression in myeloid leukemia [137]. It also interacts with c-KIT as shown by the assay of RNA pull-down. CCDC26 knockdown increases the level of c-KIT mRNA, up-regulates insulin-like growth factor 1 (IGF-1R) expression, resulting in IM resistance, whereas IGF-1R inhibition reversed IM resistance [138, 139]. H19 and FOXF1 adjacent non-coding developmental regulatory RNA (FENDRR) are oncogenic lncRNAs that are associated with cancer invasion, proliferation, and migration in various types of cancers [140]. Their expressions vary in GIST tissue, where H19 is 25.8-fold while FENDRR is 4.7-fold, both were in comparison to normal adjacent tissues [141]. Highly positive correlations between H19 and ETV1 were found in GIST cells, maybe via MEK and ERK pathways, as detected in colorectal cancer [142], and the mechanism underlining the regulation of FENDRR on the expression of KIT is not yet fully explored (Table 2).

**Table 2:** Non-coding RNA associated with c-KIT

ncRNA	Cell lines	Mechanism of action	Function	Ref
miR-218	H1975, A549, GIST882, GIST48, GIST-T1, GIST430	Targeting IL-6/STAT3 signaling pathway, Targeting PI3K/AKT signaling pathway	Suppress	[122–125]
miR-148b-3p	GIST882	Directly binding to the 3’-UTR of the KIT mRNA	Suppress	[48, 126]
miR-221/222	TF-1, HL60, GIST882, GIST48, GIST-T1	Targeting KIT/AKT signaling pathway Directly binding to the 3’-UTR of the KIT mRNA	Suppress	[47, 127–129]
miR-142-5p	GIST882, GIST-T1			[131]
miR-9				[132]
miR-370				
miR-501				
miR-494	GIST430, GIST882	Downregulate surviving (BIRC5)	Suppress	[133]
miR17-92	GIST882, GIST-T1	Directly binding to the 3’-UTR of the KIT mRNA Downregulate ETV1	Suppress	[134]
miR-20a				
miR-375-3p	GIST-T1	Directly binding to the 3’-UTR of the KIT mRNA Downregulate ETV1		[135]
miR-200b-3p				
CCDC26	HL60, GIST882, GIST-T1		Suppress	[137–139]
H19				[141]
FENDRR				

### Translation and post-translation regulation

**Fig. 2:**
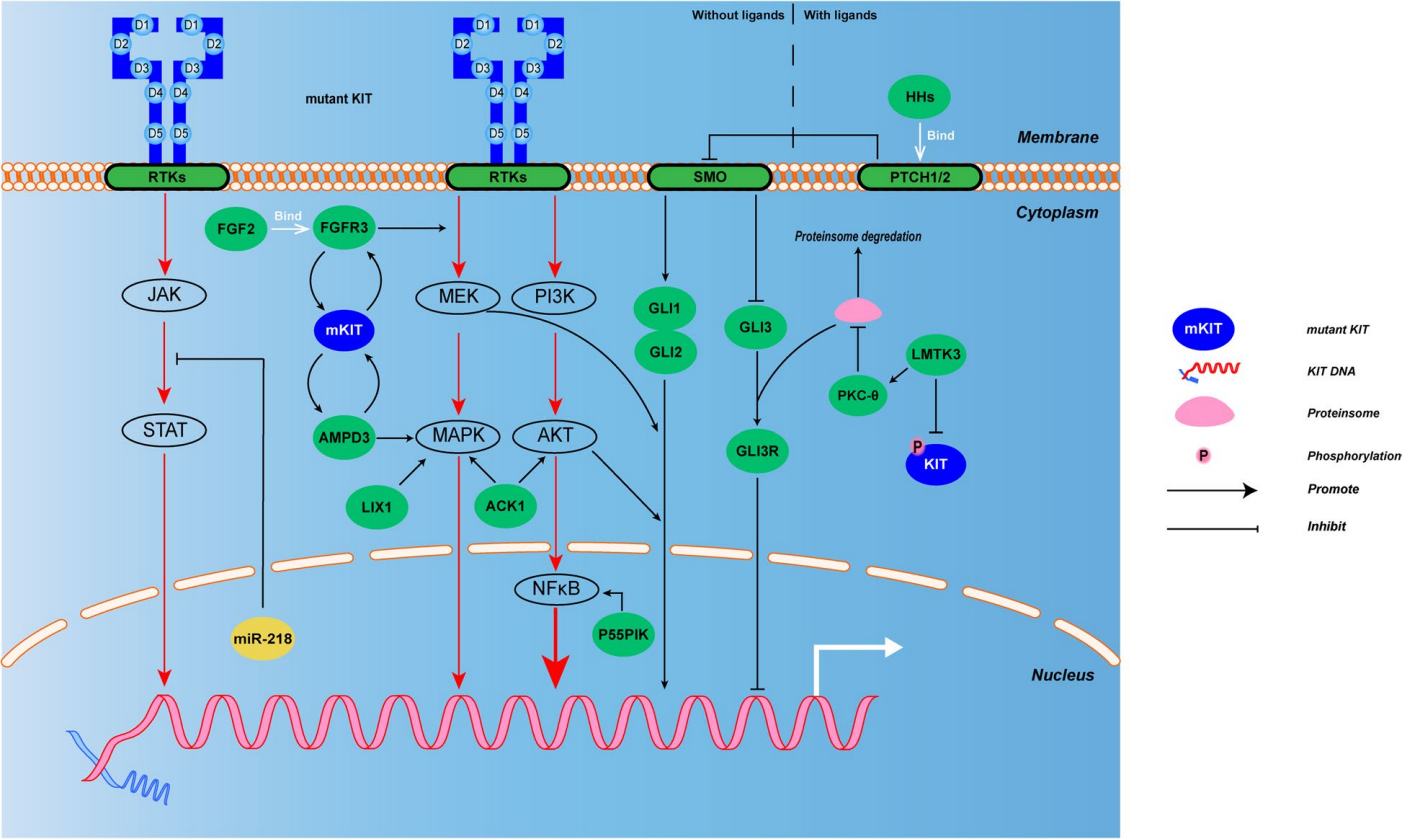
Regulations in KIT protein and signaling pathway. LMTK3 directly promotes KIT protein translation, but also inhibit the expression of PKC (PKC-θ) and KIT phosphorylation. LIX1 controls MAPK pathway. Hedgehog pathway has two activate models: with ligands, HHs binding to the receptors, Patched-1/2, initiate the Hh pathway and inhibit SMO; without ligands, SMO starts to activate different GLIs, GLI1/2 up-regulate the KIT mRNA level, while GLI3 down-regulates KIT mRNA levels via the proteasome pathway. There are three classic intracellular signaling pathways for KIT activation, respectively Ras-Erk pathway, PI3K-AKT pathway and JAK-STAT pathway. Ras-Erk pathway and PI3K-AKT pathway have crosstalk with Hh pathway. FGF2 binds to and activates its receptors FGFR (FGFR3), mediating the reactivation of mutant KIT and JAK-STAT pathway. AMPD3 also promotes the JAK-STAT pathway. FGFR3 and AMPD3 both form a positive protein–protein feedback loop with mutant KIT. MiRNA-218 can inhibit JAK-STAT pathway and P55PIK can promote PI3K-AKT pathway. ACK1 activates the MAPK pathway and the PI3K pathway, whereas lix1 may only regulates the MAPK pathway

The vast majority (95%) of GIST express KIT protein which is usually higher in the GIST with KIT mutations than those without KIT mutations [143]. Upon the IM treatment, c-KIT oncoprotein was substantially up-regulated, hinting as a protective mechanism for GIST cells to escape from TKIs, which was widely accepted as one of the major resistance mechanisms [144] (Fig. 2).

### Protein translation rate

Protein translation was another rate-limiting process of KIT expression. Lemur tyrosine kinase-3 (LMTK3) is a kind of serine/threonine kinase that regulates genes transcription, translation, and the stability of proteins via chromatin condensation and binding of the chromatin to the nuclear periphery, or tethers to DNA region for its function as tumorigenesis-promoting [145, 146]. Intriguingly, LMTK3 specifically modulates KIT gene-specific translation in GIST and melanoma cells, but not in the mast cell line and primary leukemia. LMTK3 also accelerates the translation rate of the KIT gene, resulting in secondary mutations that further leads to KIT resistance to IM [147, 148].

### Protein translocation and stabilization

The auto-phosphorylation of KIT mutant is spatiotemporally regulated. The fully transformed products of KIT mutants, KIT onco-proteins were usually in the endoplasmic reticulum, transferring to the Golgi apparatus and activated as an immature KIT protein form [149–151]. Protein kinase C (PKC) is a superfamily characteristic of phospholipid-dependent serine-threonine kinases. It physiologically regulates cell stability and differentiation. As one member of PKC, Protein kinase C-θ (PKC-θ) is highly expressed in the ICCs of the digestive tracts of guinea pigs, and in GIST ranging from 72 to 98% [152]. PKC-θ promotes the Golgi complex retention of mutant-KITs and blocks its proteasomal degradation, thus sustains the activation of abnormally localized intracellular activation of MT-KITs [151, 153]. PKC-θ over-expression was significantly positively correlated with KIT expression and poor clinicopathological characteristics and worse prognosis [154].

### Signal pathways involved in KIT oncogenesis and expression

Mutations on KIT often result in abnormal activation of kinases that activate downstream MAPK, STATs and PI3K pathways, promoting tumorigenesis and malignant progression [155, 156].

### Hedgehog signaling pathway

The genes correlated with the Hedgehog (Hh) pathway are robustly expressed in ICC stem cells and mature cells of human and murine intestines, indicating its indispensable role in the development of epithelium and mesenchymal cells in GI tract. Physiologically, the ligands (HHs), sonic Hedgehog (Shh), Indian Hedgehog (Ihh), and Desert Hedgehog (Dhh), bind to the receptors, Patched-1 (Ptch-1) and Patched-2 (Ptch-2), respectively, thereby initiating the Hh pathway [157, 158]. Loss of the 7-pass transmembrane protein Smoothened (SMO) inhibition leads to the regulation of Hh pathway which controls the expression levels of KIT mRNA via its downstream glioma-associated oncogene homolog isoform1, 2, and 3(GLI1, 2, or 3) [159]. In the absence of ligand, SMO starts to activate the hedgehog pathway [160]. Various GLI1, 2, and 3 have different roles in the expression of KIT mRNA. GLI1/2 up-regulate the KIT mRNA level, while GLI3 is a transcriptional repressor on KIT mRNA levels via the proteasome pathway [161]. Besides, the Hh pathway has crosstalk with PI3K/AKT/mTOR and RAF/MAPK/ERK signal cascades involved in the KIT regulation [162]. In vivo, targeting the Hh pathway can reduce KIT mRNA and re-enhance the sensitivity of GIST cells to TKIs.

### PI3K signaling pathway

PI3K pathway is the dominant signal directly engaged by mutant KIT oncogenic cascade in GIST, therefore, PI3K inhibitors showed meaningful efficacy when combined with IM is ongoing in the clinical trials for the treatment of GIST [163]. In mutant KITs, they direct active themselves and engaging PI3K pathway to promote imatinib resistance. For example, in double-mutant KitV558Δ; Y567F/Y567F knock-in mice which lack the SRC family kinase-binding site on KIT (pY567) exhibited attenuated MAPK signaling, and engagement of the PI3K pathway for tumor growth [164], as shown in Table 1. Phosphoinositide-3-kinase, regulatory subunit 3 (gamma) (PIK3R3, p55PIK) is a regulatory subunit of phosphoinositide 3-kinase (PI3K) and is involved in ICC hyperplasia which is prone to the tumorigenesis of GIST [32, 165]. Over-expression of p55PIK in GIST882 cells bind the promoter and increase the expression of NF-κB p65 (Ser536), leading to resultant KIT upregulation. Similar mechanism of p55PIK activating the NF-κB signal was also observed in the colorectal cancer cells [166]. Non-RTK activated CDC42 associated kinase 1 (ACK1), was colocalized and form complex with KIT protein in GIST cells and ACK1 activation is in a partially KIT and CDC42 dependent manner. Treatment with a specific ACK1 inhibitor AIM-100 or ACK1 siRNA markedly inhibits cell migration in imatinib sensitive and in imatinib resistant GIST cell lines, which is associated with inactivation of PI3K/AKT/mTOR and RAF/MAPK signaling pathways [167]. Recently, a study of PD-1/PD-L1 blockade rescue exhausted CD8 + T cells via the PI3K/Akt/mTOR signaling pathway in GIST revealing the involvement of PI3K in immunotherapy in GIST [19].

### FGF2- FGFR3 and JAK/ STAT signaling pathway

Fibroblast growth factors (FGFs) and their receptors (FGFR1-4) are universally expressed in human tissues and have an important effect on various cell physiologic processes ranging from cell proliferation, survival, and migration. The dysregulation of the FGFs signal pathway is extensively involved in several types of cancers [168]. FGF2 is highly expressed in Imatinib-resistant GIST cells and the tumor tissue from the patients who progressed on Imatinib [169]. FGF2 binds to and activates its receptors FGFR, mediating the reactivation of KIT and MAPK pathways [170]. The opening of CTCF binding in the chromosome topology of FGF and KIT mentioned above leads to the increase of their expression, which may demonstrate the close relationship between FGF and KIT in the development of resistance in GIST. The Janus kinase/signal transducers and activators of transcription (JAK/ STAT) pathway is one of the important downstream pathways by KIT activating [171]. KIT D816V and KIT N882 both are situated closely on the KIT receptor activation loop activating the Janus kinase/signal transducers and activators of transcription (JAK/STAT) pathway, but in KIT N822K it is also the downstream activation of the MAPK [172]. The V558Δ; V653A mutant mislocalization of Golgi, and lead to the enhanced activation of STAT3 and STAT5, although no differences were seen in MAPK or PI3K pathway activation, therefore, contributing to the increased tumor oncogenesis, compared to control mice with a single V558Δ Kit mutation. Blocking KIT’s localization to the Golgi from the endoplasmic reticulum (ER) can inhibit oncogenic signaling [173].

### MAPK signaling pathway

AMPD3, belonging to the adenosine monophosphate deaminases (AMPDs), functions as a main catalyzer which hydrolytically deaminates AMP to inosine monophosphate. This is a vital step in nucleotide metabolism aiding and energy balance in cells. AMPD3 is extensively expressed in tumor tissues, and its knockdown may favor the activation of AMPK, and subsequently result in the inhibition of the anabolic pathways which is prerequisite for the survival of cancer cell [174]. AMPD3 is significantly related to KIT expression in GIST. Interestingly, when the expression of KIT or AMPD3 was suppressed by siRNA, respectively, the expression of AMPD3 or KIT was also comparably decreased. These results indicate that KIT and AMPD3 may form a positive protein–protein feedback loop to promote their reciprocal expression, albeit their underlying mechanism remaining unknown [175]. Limb Expression 1 (LIX1) is a unique marker of digestive mesenchyme immaturity. It regulates mesenchymal progenitor proliferation and differentiation by controlling the Hippo effector Yes-associated protein 1 (YAP1), which is constitutively activated in many sarcomas [176, 177]. In GIST, upon LIX1 inactivation in GIST cells, YAP1/TAZ activity is reduced, KIT, as the GIST signature, is down-regulated via reducing YAP1/TAZ protein level, and cells acquire smooth muscle lineage features [178]. Moreover, LIX1 can controls MAPK signaling pathway [179]. Upon the condition of hypoxia during IM treatment in GIST, hypoxia inducible factor 1 alpha (HIF-1α) can increase the transcription level of MET gene via its bind to the MET promoter [180]. Ligation of upregulated MET by hepatocyte growth factor (HGF) expand the activation of the downstream of mitogen-activated protein kinase (MAPK). And the latter then stabilizes ETV1 for promoting KIT expression [181, 182]. These results strongly suggested that reactivation of MAPK by bypass signal may represent a therapeutic vulnerability for targeting KIT expression.

### Targeting KIT expression and mutation in GIST

**Fig. 3:**
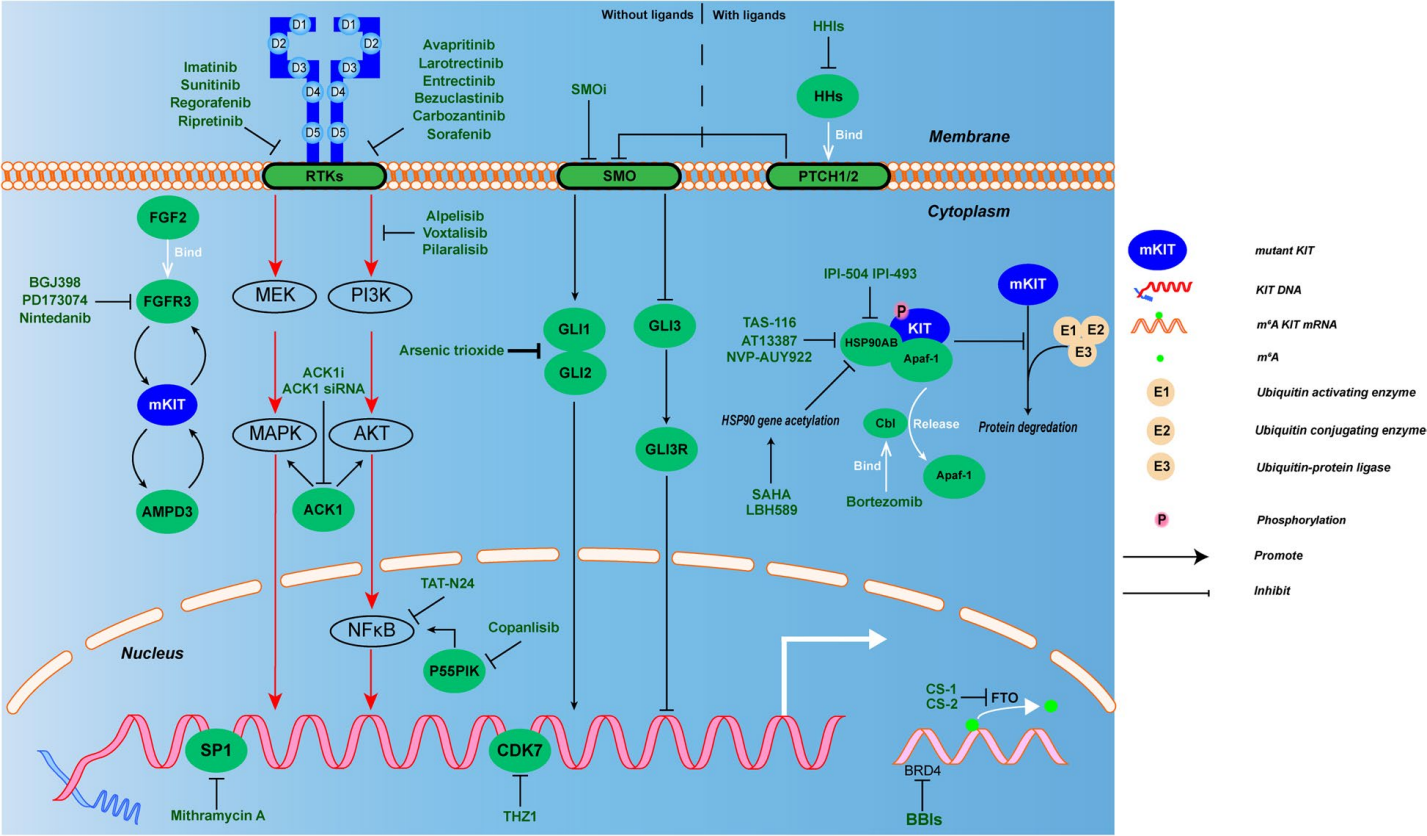
The mechanism of action of existing drugs targeting KIT expression and mutations. In this figure we describe several inhibitors targeting KIT expression and function. Therapeutic strategies of TKIs targeting KIT mutations in gastrointestinal stromal tumors: fist-line therapy-Imatinib; second-line therapy-Sunitinib; third-line therapy-Regorafenib; fourth-line therapy-Ripretinib. Other new drugs targeting KIT mutations in gastrointestinal stromal tumors: Avapritinib, Larotrectinib, Entrectinib, Bezuclastinib, Carbozantinib, Sorafenib. The c-KIT-Hsp90Β-Apaf-1 complex inhibits the ubiquitination degradation of mutant KIT. Bortezomib binds to Cbl, destabilizing the c-KIT-Hsp90Β-Apaf-1 complex and releasing Apaf-1, and then KIT proteins are internalized and degraded in GIST cells. IPI-504, IPI-493, TAS-116, AT13387 and NVP-AUY922 are HSP90 inhibitors. HDAC inhibitors, SAHA and LBH589, attenuate the activity of HSP90 by acetylating on HSP90 gene. For Hh pathway, HHs, SMO and GI1/2 have their own inhibitors. PI3K/mTOR inhibitor voxtalisib, the pan-PI3K inhibitor pilaralisib, and the PI3K-restricted inhibitor alpelisib all reducing GIST cell proliferation. TAT-N24 and the emerging PI3K or P55PIK inhibitors, such as Copanlisib, both inhibit NF -κB. BGJ398, PD173074 and nintedanib are FGFR inhibitor targeting FGFR1-4. ACK1 inhibitor AIM-100 or ACK1 siRNA inhibits ACK1. CS-1 and CS-2 are functioned as FTO inhibitors preventing KIT m6A mRNA demethylation. BBIs can reverse the transcription abnormalities of KIT gene which are induced by BRD4. Mithramycin A inhibits the TF, SP1, and HZ1 decreases the transcriptions of OSR1

In screening a compound library enriched for U.S FDA-approved chemotherapeutic agents, GIST cells displayed high sensitivity to transcriptional inhibitors, and mechanistically, these compounds exploited the cells dependency on continuous KIT expression. For example, Mithramycin A inhibits the TF, SP1, which is also a major transcriptional activation of the KIT gene, thus explaining why Mithramycin A induces apoptosis [183]. These indicated that it is plausible to target KIT abnormal regulatory-circus, together with kinase activity-inhibition in GIST treatment (Fig. 3).

### Targeting special transcriptional factors

Clinically, both HAND and BARX1 can be positive predictors for the progression or relapse events in the patients with metastatic GIST or post-operation GIST, respectively. HAND was enriched in small intestine ICCs while BARX1 was enriched in micro-GIST [35], thereby offering site-dependent targets for GIST treatment. In vivo, ETV1 is a critical survival factor for the growth of Imatinib-sensitive and Imatinib-resistant GIST cell lines [70]. Double inhibition of MAP kinase and KIT signaling can synergistically destabilize ETV1 by interrupting the positive feedback loop of KIT-MEK-ETV1-KIT. Recent Phase II clinical trial showed an overall response of 69.0% and median progression free survival of 29.9 ms in the treatment-naïve patients with advanced GIST using MEK inhibitor Binimetinib plus Imatinib [184, 185]. In addition, SHBP2 and FOXF1 could be an ideal target for being further explored therapeutically in the treatment of GIST.

Phenothiazine is a cytotoxic drug that induces apoptosis and autophagy that showed synergistic role with ERK-inhibitor in the treatment of GIST [186]. CDK7 mRNA and protein levels are elevated in high-risk GIST and indicated of poor clinical outcomes, in which CDK7 is a key activator of RNAPII that preferentially dysregulate RNAPII CTD phosphorylation. THZ1 blocks the role of CDK7 on RNAPII, and thus decrease the transcriptions of odd-skipped related transcription factor 1(OSR1), which is one of super enhancer (SE) of KIT gene in GIST [187]. Similarly, KIT-regulated enhancer domain in GIST could be targeted by BRD4, a crucial activator of RNAPII transcription at active chromatin marks, and the BBIs can reverse the transcription abnormalities of targeted genes which are induced by BRD4 [67, 188].

### Targeting epigenetic modification molecules

The combination of BBI and TKI led to superior cytotoxic effects in vitro and in vivo, with the advantage of preventing tumor growth in TKI-resistant GIST xenografts. The expression profiles of GIST cells treated with BBIs were akin to those treated with KIT inhibition, indicating the KIT expression be the target of BBIs [113]. Also, BET bromodomain is becoming an attractive target for KIT-mutant GIST as shown in clinical trials. A synthetic analogue of cerulenin, JQ1, has been shown to potently inhibit BRD4 and exhibited synergy with imatinib and induced apoptosis and autophagy in vitro [188].

Epi-transcriptional modifications on mRNA mainly influence its stability and expression, several molecules function as “writers” or “erasers” for the regulation of mRNA. Our previous study showed that FTO inhibition can dramatically inhibit cell proliferation and increase sensitivity of GIST cells to IM-induced apoptosis. CS-1 and CS-2 were recently repurposed as a FTO inhibitor and exhibited the synergistic effect with IM in KIT-mutated GIST [189, 190].

### Targeting ncRNA

With the successful development of mRNA COVID-19 vaccines and the approval of a number of novel RNA-based drugs, RNA has jumped to the forefront of drug research [191]. In addition to mRNA's role in producing antigens or therapeutic proteins, different types of RNA have a variety of functions and play important regulatory roles in cells and tissues. One type of these RNAs, lncRNAs, has the potential to be used as new therapies, and the lncRNA itself can be used as both drugs and targets. To date, no studies have associated lncRNA treatment to altered expression of KIT gene and mutations in GIST, despite the fact that some lncRNAs that have been shown to regulate KIT expression in GIST and are associated with imatinib resistance [132, 192]. For example, miRNA sponges is a kind of artificial transcripts that contains multiple miRNA binding sites to trap and sequester it [193]. Although this tool can simultaneously inhibit miR-221/miR-222 in tumour cells such as breast cancer cells [194] and recently a PhaseI study of the frst-in-class locked nucleic acid (LNA) miR-221 selective inhibitor shows good safety and SD + PR in refractory advanced cancer patients [195]. The above suggests promising therapeutic ability in targeting ncRNA and thereby modulating KIT expression warranted future investigation. And intriguingly, use of an aptamer-based method for KIT expression targeted detection of GIST was successfully executed in vitro and in vivo, and intracellular delivery of TKIs to signaling terrace via anti-KIT DNA aptamer or modified RNA aptamers to inhibit the activity of KIT kinase, heralding a novel avenue in GIST treatment [196, 197].

### Targeting protein translation and post-translation process

LMTK3 increases KIT expression via the speedup of translation rate of KIT transcripts. LMTK3 knockdown not only directly decreases KIT protein translation, but also inhibit the expression of PKC and KIT phosphorylation, indicating LMTK3 as a druggable target. Mutated KIT protein is usually detained within intracellular organelle and chemical inhibition for intracellular transport to Golgi or specific organelles-targeting TKIs by chemical modifications seems a promising therapeutic strategy [198]. Intracellular delivery of TKIs to signaling terrace via anti-KIT DNA aptamer or modified RNA aptamers to inhibit the activity of KIT kinase, heralding a novel avenue in GIST treatment [196, 199, 200]. Homoharringtonine (HHT) is a first-in-class inhibitor of protein biosynthesis and is FDA-approved for the treatment of chronic myeloid leukemia (CML) that is resistance to at least two lines of treatment with TKIs. In vivo and in vitro experiment, HHT showed significant anti-proliferation and apoptosis-induction with a notable reduction of KIT protein and subsequent decrease of KIT activation and downstream signaling, while KIT mRNA levels were slightly affected [201].

Bortezomib is a dipeptide boronic acid inhibitor of the 20S proteasome. It synergistically augmentes the efficacy of TKIs in multiple myeloma and mantle cell lymphoma. Bortezomib can bind to Cbl, an E3 ubiquitin-protein ligase, destabilizing the c-KIT-Hsp90Β-Apaf-1 complex and releasing Apaf-1; then KIT was unleashed from the complex, and be internalized and degraded in the cytoplasm of GIST cells. Treatment with bortezomib can enhance dasatinib-induced apoptosis in GIST T1 cells and increase the inhibition effect of IM on cell proliferation and invasion in GIST 882 cells[144, 202]. HSP (heat-shock protein) is a multi-protein chaperone, acting as a key mediator for the correct folding, intracellular disposition, and proteolytic turnover of the proteins which are responsible for cell growth and survival. The fundamental chaperoning role of HSPs is subverted, especially heat-shock protein of 90 KD (HSP 90), leading to the maintenance of mutant proteins with its gain-of-function of protecting cancer cells from onco-stress [203].

HSP 90 inhibitors, IPI-504 and AT13387 can decrease the KIT protein level, showing obvious antitumor activity in GIST as a single agent, and they are more potent when in combination with IM or sunitinib [54, 204]. HSP90AA1, one of the client proteins of HSP90, is a major chaperone protein for KIT oncoprotein with a protective role from its degradation in GIST. Other HSP90 inhibitors, including 7-allylamino-17- demethoxygeldanamycin (17-AAG) and IPI-493, also exhibit the inhibition effect in cell growth due to the degradation of mutant KIT protein via both proteasome- and autophagy-dependent pathways [53]. Novel HSP90 inhibitors, NVP-AUY922 and TAS-116, can downregulate both total and phosphorylated KIT proteins, and mTOR inhibitors can enhance the inhibitory role of NVP-AUY922 in GIST cells [205–207]. Intriguingly, the HDAC inhibitors (HDACi), SAHA and LBH589 can epigenetically attenuate the activity of HSP90 by its acetylating on HSP90 gene, with the resultant degradation of KIT protein. In vitro and vivo experiments, HDAC inhibitors presented with a synergistic effect in the IM-treated GIST cells [208]. Although there are many drugs directly targeting HSP 90 on its co-factors, challenges remain in clinical translation for its endurable toxicity [209]. Genome-wide functional screening identifies CDC37 as a crucial HSP90-cofactor for KIT oncogenic expression in gastrointestinal stromal tumors [210]. PBOX-15, a novel microtubule-targeting agent (MTA) reduced CKII expression, an enzyme which regulates the expression of CDC37, which downregulate KIT expression via CDC37-HSP90 mediated KIT degradation [211]. These findings indicate the potential of PBOX-15 to improve the apoptotic response of IM in GIST cells and provide a more effective treatment option for GIST patients.

### Targeting signal pathways regulating KIT expression

Targeting the Hh pathway can reduce KIT mRNA and re-enhance the sensitivity of GIST cells to TKIs in in vivo experiments. GI1/2 inhibitor, arsenic trioxide, decreases KIT expression and reduces cell viability by significantly increasing the bonding of GLI3 to the KIT promoter, demonstrating efficacy in the Imatinib-sensitive and Imatinib-resistant GIST cells [157]. The p55PIK specific inhibitor, TAT-N24, can abrogate the resistance of GIST cells to Imatinib [32] and dramatically down-regulated KIT expression and enhanced the Imatinib effectiveness in an NF-κB -dependent manner as validated in the PDX tissue from IM-resistance-GIST patients. Inhibitors of the PI3K pathway have already made significant contributions to GIST, with the dual PI3K/mTOR inhibitor voxtalisib, the pan-PI3K inhibitor pilaralisib, and the PI3K-restricted inhibitor alpelisib all reducing GIST cell proliferation [164]. The emerging PI3K or P55PIK inhibitors, including Copanlisib, showed high efficacy and low toxicity in IM-resistant GIST [165, 212].

KIT and FGFR3 have a direct interaction in GIST cells, and BGJ398, a selective FGFR1-4 inhibitor, can re-sensitize GIST to IM in in vitro and in vivo experiments [170]. With the combination of BGJ398 and sunitinib, SDH-GIST patients may get better outcomes [102]. Nintedanib, first approved by FDA for idiopathic pulmonary fibrosis, overcame not only resistance induced by KIT mutations, but also ERK-reactivation-mediated resistance induced by FGF upregulation [43]. The combination of FGFR inhibitor PD173074 and IM showed highly synergistic effect in IM-resistant GIST cells [213]. The phase Ib study of BGJ398 and imatinib in the treatment of IM-refractory advanced GIST showed that the primary endpoint was achieved in which approximately 25% (3/12) of patients sustained stable disease for more than 32 weeks [214]. That suggests that FGF inhibitors like BGJ398 might be a promising treatment strategy combination with TKIs after imatinib resistance.

### Targeting KIT mutations

Different genotypes of KIT mutations are associated with diversified responses to specific TKIs. Primary mutations often appear in KIT Exon 9 and 11, and the latter is more sensitive to Imatinib with better progression-free survival (PFS), and median overall survival (OS). However, IM showed almost ineffective for secondary KIT mutations including exon 13, 14 and exon 17, 18 [215, 216]. In the setting of second and beyond, sunitinib had better inhibitory effect on KIT mutants with exon 9 or 11/13 or 14 double mutations than IM, but regorafenib, dasatinib, nilotinib and sorafenib was largely effective to inhibit the phosphorylation of KIT with secondary exon 17 mutation [217–219]. In animal model, compared to control mice with a single V558Δ Kit mutation, mice with a double V558Δ; V653A Kit mutation had increased tumor oncogenesis and associated KIT-dependent STAT activation, while cabozantinib was more effective in overcoming resistance than sunitinib[125]. In vitro, V654A mutation encoded by KIT exon 13 was intermediately sensitive to anlotinib. Moreover, anlotinib was able to partly suppress the activation loop mutation D820A from exon 17 while another activation loop mutation N822K, also from exon 17, was resistant to anlotinib [220]. BLU-263 (avapritinib), targeting KIT D816V, is currently being evaluated in the phase 2/3 HARBOR study of patients with ISM [221]. Recently, PLX-9486 (Bezuclastinib), an active-state TK inhibitor with activity against mutations in KIT exons 9, 11, 17, and 18, including D816V, has several clinical trials and shows great clinical benefit with an acceptable safety profile either using alone or in combination with other TKIs [222, 223].

## Conclusion and perspective

Collectively, continued KIT-dependency is a typical characteristic of GIST, and complete tumor eradication of non-operable GIST may require a powerful inhibition of the KIT pathway, which is potentially attained by targeting both the tyrosine kinase and the abnormal overexpression of KIT protein. Currently, seven drugs targeting KIT mutations have already been approved by the FDA for the treatment of advanced-stage GIST (imatinib, sunitinib, regorafenib, ripretinib, avapritinib, larotrectinib and entrectinib), all of which are TKIs [22]. Although these agents can be very effective for treating certain GIST subtypes, challenges remain and novel therapeutic approaches are needed [12, 224]. GIST is inherently resistant to radiotherapy and cytotoxic drug [225], albeit some end-stage cases with GIST treated by radiotherapy can achieved palliative pain-relief [226–228]. Immunotherapy is a promising hotspot in the treatment of tumors [229, 230], but the efficacy cannot be determined in the existing cases of GIST [231]. Efforts continue to be made in immunotherapy as well as radiation therapy for GIST [232–235].

It has been speculated that special regulation network exclusively in KIT-driven GIST, on the gene coding, epigenetic modification, and protein level. Available evidence that the decrease of KIT expression via HSP90- chaperone protein for the reversal of IM resistance has become a realistic possibility in clinical application [236, 237]. This fact further improves KIT value not only as a diagnosis marker, but also could be a predictive marker for the therapeutic treatments targeting KIT expression. Thus, the development of standardized approaches to measure KIT expression in different molecule level or various cellular organelles will make it possible.

The emerging molecules which were unravelled recently such as FOFX1, ETV1, which have potentially sculpted the innate link of GIST and its derivation ICC. Because ETV1 also regulates the expression of ICC gene signatures and is crucial for ICC cell survival, as well as its upstream FOFX1 do [74]. So deeply exploring the key gene expression and regulation process of ICC lineage-specific differentiation or the compelling molecules impact its physical function such as gut motility, may offer the possibility of novel promising targets. Additionally, these KIT auto-regulation positive loop mediated by the components of the PI3K or MAPK signal pathways strengthened the interest in the conjoined inhibition of these two pathways as potential therapeutic strategy, thus support the continuous endeavor for the development of novel drugs in this field in the setting of IM-resistant GIST, although how to decrease the toxicity of these combination is a key unanswered question.

The crosstalk between various RTK is the common phenomenon in cell body, this KIT in GIST is not an exception. Evidence chains range from basic research to pre- or early-phase clinical trials consolidated the knowledge that FGF/FGFR signal pathway is a vital target for KIT–mutated GIST via multifaceted mechanism including decreased KIT expression, expected for large-size random clinical trial to confirm and propel FGFR-targeted therapy in GIST patients in the future.

**Fig. 4:**
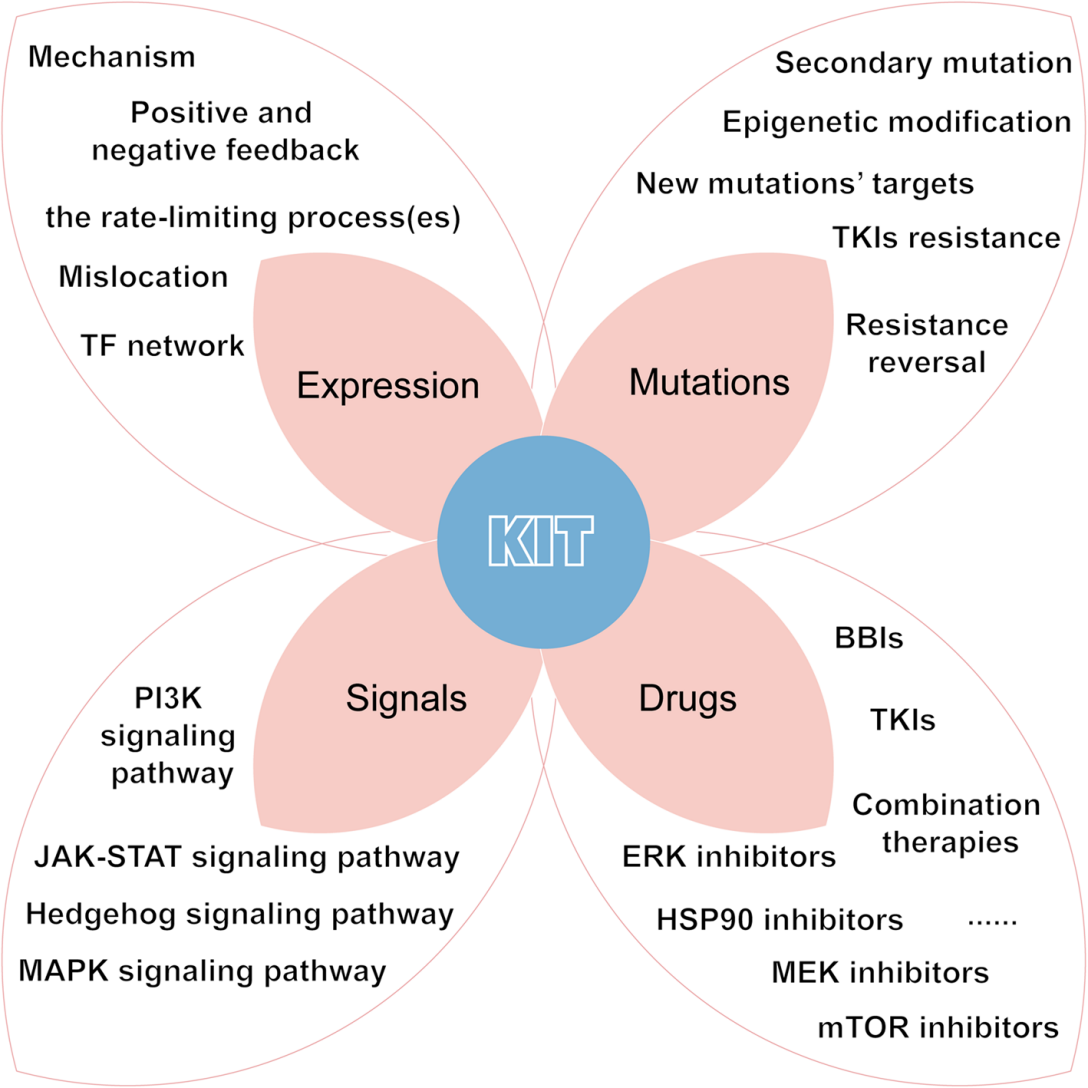
Perspective of KIT in GIST. KIT expression and its downstream activation pathway regulation mechanism needs to be taken seriously as its mechanism is not fully explained. KIT mutations are the main culprit responsible for the development of GIST and the primary/secondary resistance of imatinib. The epigenetic modification of KIT mutation and the study of new mutations’ targets are worthy of further study. Further treatment options may consider combining TKIs with other drugs that target KIT and their signaling pathway to achieve better efficacy and address drug use in resistant patients

Taken together, there is no doubt that KIT oncoprotein remains a therapeutic target for novel drug or drug-repurpose because the heterogeneous mutations within KIT oncogene generated for the reactivation of KIT signal cascade, contributing to the disease progression in most cases after the failure of IM and even multiple-lines of TKIs. Current research is evaluating the agents target the high level of KIT expression, either alone or in combination with imatinib or other TKIs, aiming to circumvent the high rate of resistance. However, several research questions remain unanswered including (Fig. 4) (Table 3):

- what is the relationship between KIT activation and KIT upregulation, the rate-limiting processes for abnormal KIT expression in GIST,
- if the underlying mechanism of KIT expression is exclusive to GIST, but not in WT KIT,
- imatinib treatment induces the change of tumor microenvironment (TME) [231, 238], if it in turn affected the expression of KIT gene,
- upon the IM treatment, c-KIT oncoprotein was substantially up-regulated, which was considered as a protective mechanism for GIST cells, if these from the release of negative feedback, such as sprouty homolog 4 (SPRY4) [239],
- the protein of KIT mutants usually mislocated within intracellular organelles, such as golgi apparatus, how to detect them used for the guidelines of drugs targeting KIT expression?

**Table 3:** Clinical trials of the drugs targeting KIT expression in GIST

Drug	Category	Combination	Phase	Status	Sponsor location	NCT Number
Entacapone	FTO demethylation inhibitor	Imatinib	1	Active, not recruiting	China	04006769
BBI503	BRD4	/	2	Terminated	Canada	02232620
BBI503	BRD4	/	1	Completed	US	02354898
BIIB021	HSP90 inhibitor	/	2	Completed	US	00618319
IPI-504	HSP90 inhibitor	/	1	Completed	US	00276302
Pimitespib	HSP90 inhibitor	Imatinib	1	Recruiting	Japan	05245968
AT13387	HSP90 inhibitor	Imatinib	2	Completed	US	01294202
Ganetespib	HSP90 inhibitor	/	2	Completed	US	01039519
AUY922	HSP90 inhibitor	/	2	Unknown	Taiwan, China	01389583
AUY922	HSP90 inhibitor	/	2	Completed	US	01404650
IPI-504	HSP90 inhibitor	/	3	Terminated	US	00688766
BKM120	PI3K inhibitor	Imatinib	1	Completed	US	01468688
BYL719	PI3Kα inhibitor	Imatinib	1	Completed	US	01735968
Copanlisib	PI3K inhibitor	BAY1895344	1	Not recruiting	US	05010096
Perifosine	AKT inhibitor	Imatinib	2	Completed	US	00455559
Perifosine	AKT inhibitor	Sunitinib	1	Completed	Canada	00399152
RAD001 (Everolimus)	mTOR inhibitor	Imatinib	1/2	Completed	US	01275222
RAD001 (Everolimus)	mTOR inhibitor	/	/	Available	/	03493152
Temsirolimus	mTOR inhibitor	/	/	Recruiting	Germany	00700258
RAD001 (Everolimus)	mTOR inhibitor	/	2	Completed	Germany	00767819
MEK162 (Binimetinib)	MEK inhibitor	Ripretinib	1/2	Withdrawn	US	05080621
MEK162 (Binimetinib)	MEK inhibitor	Pexidartinib	1	Completed	US	03158103
MEK162 (Binimetinib)	MEK inhibitor	Imatinib	1/2	Active, not recruiting	US	01991379
MEK162 (Binimetinib)	MEK inhibitor	Ripretinib	1/2	Withdrawn	Unknown	05080621
Trametinib	MEK inhibitor	Pazopanib	2	Withdrawn	US	02342600
Selumetinib (AZD6244)	MEK inhibitor	/	2	Withdrawn	US	03109301
Sorafenib	Raf inhibitor	/	2	Completed	Korea	01091207
Sorafenib Tosylate	Raf inhibitor	/	2	Active, not recruiting	US	00265798
THE-630	Pan-KIT inhibitor	/	1/2	Recruiting	US	05160168
Linsitinib	IGF-1R inhibitor	/	2	Completed	US	01560260
BGJ398	Pan-FGFR inhibitor	Imatinib	1b	Completed	US	02257541
Vismodegib	Hedgehog inhibitor	/	1/2	Completed	US	01154452
Arsenic trioxide	GI1/2	/	1	Completed	US	00124605
Arsenic trioxide	GI1/2	/	1	Completed	US	00003630
XL820	GLIPR1 inhibitor	/	2	Completed	US	00570635
Vorinostat	HDACI	/	2	Completed	Germany	00918489
LBH589	HDACI	/	2	Completed	France	01136499
Selinexor	Selective inhibitor of Nuclear Export	Imatinib	1/2	Recruiting	Spain	04138381
BLU-263	KIT Exon17 D816V	/	2/3	Recruiting	US	04910685
Bezuclastinib	KIT exons 9, 11, 17, and 18, including D816V	/	2	Recruiting	US	04996875
Bezuclastinib	KIT exons 9, 11, 17, and 18, including D816V	/	2/3	Recruiting	US	05186753
Bezuclastinib	KIT exons 9, 11, 17, and 18, including D816V	Pexidartinib/ Sunitinib	1b/2a	Completed	US	02401815

## Data Availability

Not applicable.

## Abbreviations

TK: Tyrosine kinase
RTK: Receptor tyrosine kinases
SCF: Stem cell factor
ICC: The interstitial cell of Cajal
GIST: Gastrointestinal stromal tumor
PGFRA: Platelet-derived growth factor receptor α
SDH: Succinate dehydrogenase
NF1: Neurofibromin 1
KRAS: Kirsten rat sarcoma viral oncogene homolog
HRAS: Harvey rat sarcoma viral oncogene homolog
JM: Juxtamembrane
IM: Imatinib
TKIs: Tyrosine kinase inhibitors
WT: Wild type
IHC: Immunohistochemistry
CREs: Cis-regulatory elements
RNA Pol II: RNA Polymerase II
TF: Transcription factor
FOXF1: Forkhead box F1
DBD: DNA binding region
ETV1: ETS translocation variant 1
MAPK: Mitogen-activated protein kinase
CREB: Cyclic AMP-responsive element-binding protein
CBP: Cyclic binding protein
HAND1: Heart and Neural Crest Derivatives Expressed 1
GPR20: G protein-coupled receptor 20
BARX1: Homeobox protein BarH-like 1
SH3BP2: C-Abl Src homology 3 domain-binding protein-2
PH: Pleckstrin homology
MITF: Microphthalmia-associated transcription factors
EEs: Epigenetic enzymes
PPM1A: Protein phosphatase 1A
KIF1B: Kinesin family member 1B
NF2: Neurofibromin 2
ENG: Endoglin
TGF-h: Transforming growth factor-h
CTCF: CCCTC-binding factor
TADs: Topologically associated domains
ANO1: Anoctamin 1
SE: Super enhancer
m^6^A: N6-methyladenosine
mRNA: Messenger RNA
METTL3: Methyltransferase-like 3
FTO: Fat mass- and obesity-associated
AML: Acute myeloid leukemia
KDM4D: Lysine (K)-specific demethylase 4D
BRD4: Bromodomain-containing protein 4
BET: Bromodomain and extra-terminal domain-containing
NFκB: Nuclear factor kappa-light-chain-enhancer of activated B cells
CCL2: C–C motif chemokine ligand 2
BBIs: BET bromodomain inhibitor
MOZ: Monocytic zinc finger
ncRNAs: Non-coding RNAs
miRNAs: Micro-RNAs
nt: Nucleotides
LncRNAs: Long non-coding RNAs
BIRC5: Survivin
CCDC26: LncRNA coiled-coil domain-containing 26
FENDRR: FOXF1 adjacent non-coding developmental regulatory RNA
LMTK3: Lemur tyrosine kinase-3
PCK: Protein kinase C
PKC-θ: Protein kinase C-θ
Hh: Hedgehog
Shh: Sonic Hedgehog
Ihh: Indian Hedgehog
Dhh: Desert Hedgehog
Ptch-1: Patched-1
Ptch-2: Patched-2
SMO: Smoothened
GLI1, 2, and 3: Homolog isoform1, 2, and 3
p55PIK: Phosphoinositide-3-kinase, regulatory subunit 3 (gamma)
ACK1: CDC42 associated kinase 1
FGFs: Fibroblast growth factors
JAK/ STAT: Janus kinase/signal transducers and activators of transcription
AMPDs: Adenosine monophosphate deaminases
LIX1: Limb Expression 1
YAP1: Yes-associated protein 1
HIF-1α: Hypoxia inducible factor 1 alpha
HGF: Hepatocyte growth factor
OSR1: Odd-skipped related transcription factor 1
LNA: Locked nucleic acid
HHT: Homoharringtonine
CML: Chronic myeloid leukemia
17-AAG: 7-Allylamino-17- demethoxygeldanamycin
HSP90: Heat Shock Protein 90
Hsp90 Β: HSP90AB
HDACi: HDAC inhibitors
PFS: Progression-free survival
OS: Overall survival
MTA: Microtubule-targeting agent
TME: Tumor microenviroment
SPRY4: Sprout homolog 4
